# Multi-domain potential biomarkers for post-traumatic stress disorder (PTSD) severity in recent trauma survivors

**DOI:** 10.1038/s41398-020-00898-z

**Published:** 2020-06-27

**Authors:** Ziv Ben-Zion, Yoav Zeevi, Nimrod Jackob Keynan, Roee Admon, Tal Kozlovski, Haggai Sharon, Pinchas Halpern, Israel Liberzon, Arieh Y. Shalev, Yoav Benjamini, Talma Hendler

**Affiliations:** 1 Sagol Brain Institute Tel-Aviv, Wohl Institute for Advanced Imaging, Tel Aviv Sourasky Medical Center, Tel-Aviv, Israel; 2 Sagol School of Neuroscience, Tel-Aviv University, Tel-Aviv, Israel; 3 Department of Statistics and Operations Research, Tel-Aviv University, Tel-Aviv, Israel; 4 Faculty of Social Sciences, School of Psychological Sciences, Tel-Aviv University, Tel-Aviv, Israel; 5 Department of Psychology, University of Haifa, Haifa, Israel; 6 Laboratory of Early Markers of Neurodegeneration (LEMON), Department of Neurology, Tel-Aviv Sourasky Medical Center, Tel-Aviv, Israel; 7 Sackler Faculty of Medicine, Tel-Aviv University, Tel-Aviv, Israel; 8 Institute of Pain Medicine, Department of Anesthesiology and Critical Care Medicine, Tel Aviv Sourasky Medical Center, Tel-Aviv, Israel; 9 Pain Management & Neuromodulation Centre, Guy’s & St Thomas’ NHS Foundation Trust, London, UK; 10 Department of Emergency Medicine, Tel Aviv Sourasky Medical Center, Tel-Aviv, Israel; 11 Department of Psychiatry, Texas A&M Health Science Center, Bryan, TX USA; 12 Department of Psychiatry, NYU Langone Medical Center, New York, NY USA

**Keywords:** Psychiatric disorders, Neuroscience, Biomarkers, Neuroscience

## Abstract

Contemporary symptom-based diagnosis of post-traumatic stress disorder (PTSD) largely overlooks related neurobehavioral mechanisms and relies entirely on subjective interpersonal reporting. Previous studies associating biomarkers with PTSD have mostly used symptom-based diagnosis as the main outcome measure, disregarding the wide variability and richness of PTSD phenotypical features. Here, we aimed to computationally derive potential biomarkers that could efficiently differentiate PTSD subtypes among recent trauma survivors. A three-staged semi-unsupervised method (“3C”) was used to firstly categorize individuals by current PTSD symptom severity, then derive clusters based on clinical features related to PTSD (e.g. anxiety and depression), and finally to classify participants’ cluster membership using objective multi-domain features. A total of 256 features were extracted from psychometrics, cognitive functioning, and both structural and functional MRI data, obtained from 101 adult civilians (age = 34.80 ± 11.95; 51 females) evaluated within 1 month of trauma exposure. The features that best differentiated cluster membership were assessed by importance analysis, classification tree, and ANOVA. Results revealed that entorhinal and rostral anterior cingulate cortices volumes (structural MRI domain), in-task amygdala’s functional connectivity with the insula and thalamus (functional MRI domain), executive function and cognitive flexibility (cognitive testing domain) best differentiated between two clusters associated with PTSD severity. Cross-validation established the results’ robustness and consistency within this sample. The neural and cognitive potential biomarkers revealed by the 3C analytics offer objective classifiers of post-traumatic morbidity shortly following trauma. They also map onto previously documented neurobehavioral mechanisms associated with PTSD and demonstrate the usefulness of standardized and objective measurements as differentiating clinical sub-classes shortly after trauma.

## Introduction

Post-traumatic stress disorder (PTSD) symptoms are commonly observed shortly after exposure to trauma, and their initial intensity is associated with a high risk of poor-recovery^1–3^. PTSD diagnostics and prognostics are currently based on reported symptoms optimally captured by structured interviews, such as the Clinician-Administered PTSD Scale (CAPS)^4^. While these psychological assessment tools show good reliability, and both construct and predictive validity, they have notable limitations. The CAPS, for example, does not capture symptoms frequently co-expressed with PTSD, such as depression and anxiety^5^. It is also only weakly linked objectively measured cognitive performance and other putative biological features^6–8^. Additionally, the CAPS solely relies on subjective interpersonal reporting precluding objective indication of the clinical status. These limitations may be responsible for the temporal instability of PTSD diagnosis^9^ and for its sub-optimal performance as a guide for long-term individualized clinical management^10^.

Cognitive functioning is one of many dimensions often overlooked in clinical evaluations of post-traumatic psychopathology. Nonetheless, numerous cognitive deficits have been associated with PTSD, including working memory, information processing speed, verbal learning, short-term and declarative memory, attention and executive functioning, response inhibition and attentional switching (see recent meta-analyses^11,12^). Adaptive cognitive functioning has been linked with resilience and a reduced likelihood of development and maintenance of PTSD symptoms^12,13^. Similarly, several brain structure and function characteristic might underlay PTSD clinical manifestations^6,14–17^, including lower hippocampal volume^18–21^ or altered activity and connectivity amygdala, insula, anterior cingulate cortex (ACC), medial prefrontal cortex (mPFC), and the dorsolateral prefrontal cortex (dlPFC); structures known to be involved in threat detection, executive function, emotion regulation, and contextual processing^6,14,22–26^. Other structural and functional abnormalities constitute putative predisposing factors for developing PTSD^14,23,27–29^.

Despite promising indications, however, these objective cognitive and neural measures have not been integrated as a routine assessment and management plan of post-traumatic psychopathology. One obstacle in the clinical translation of these findings is a poor understanding of how these indicators cluster together into PTSD clinical subtypes and thereby may better inform the use of potential interventions. Moreover, most studies associating objective biomarkers with PTSD have used the disorder’s symptom-based diagnostics as the main outcome measure, thereby overlooking the richness of phenotypical features associated with post-traumatic psychopathology such as depression and anxiety, and imposing an extraneous construct (PTSD diagnosis) as the only outcome of interest.

The limitations mentioned above call for the use of advanced computational and statistical methods that can co-evaluate wide arrays of potential biomarkers, disorder indicators, and clinical manifestations in PTSD. Machine learning methods are particularly well-suited to address such computational challenges, as they can account for the intricate interrelation of many relevant factors^30^. Indeed, the last decade has shown an exponential increase in the use of machine learning for the study of post-traumatic stress, including both supervised and unsupervised approaches^1,31–33^. While both approaches have shown varying success, supervised methods are limited by the accuracy of the prior knowledge they rely on, and unsupervised methods are limited in that subpopulations are not tied to specific questions of interest^34^. As a result, neither approaches typically discovers novel biomarkers tied to the investigator’s questions of interest. A possible solution is the use of hybrid analytic methods that combine both supervised and unsupervised approaches, which may yield more accurate disorder categories by identifying novel combinations of potential biomarkers for specific disorders^34^.

The present study evaluates multi-domain objective measurements’ ability to identify clusters, which we hypothesize may represent post-traumatic psychopathology subtypes. The term “subtypes” here accounts for different demographics, clinical sub-scales, or symptom severity. To do so, we applied a recently developed three-stage hybrid analytic methodology, termed 3C (categorize, cluster, and classify)^35^, to a dataset obtained in 101 individuals recruited from the emergency room shortly after exposure to a traumatic event. The 3C is a semi-unsupervised method that combines theory- and data-driven approaches, therefore used both subjective symptom-driven clinical knowledge (supervised) with state-of-the-art data-driven methods (unsupervised). Our dataset included objective measures obtained from neuroimaging and cognitive testing of recent trauma survivors, within 1 month following the traumatic incident. We assumed that the 3C hybrid analytic approach would unveil a unique set of potential mechanism-related cognitive and neural biomarkers for PTSD, closely tied to pre-existing diagnostic methods.

## Materials and methods

The 3C method here used a multi-domain data set composed of clinical interviews, psychometrics and questionnaires, computerized cognitive testing, structural and functional neuroimaging indices. The data were obtained from recent trauma survivors seen in a general hospital’s emergency department (ED) following traumatic events, as part of a larger project examining PTSD development in trauma survivors during the first critical year following exposure (for study protocol please see Ben-Zion et al.^36^). For this study, we used data obtained within 1 month of trauma exposure.

### Participants

A total of 101 recent trauma survivors (age = 34.80 ± 11.95, range 18–65, 51 females) admitted to ED following a traumatic experience were included in this analysis. The most common trauma type was motor vehicle accidents (*n* = 79, 78%). Other traumatic events included assaults, terror attacks, drowning, mass casualty incidents, robbery, and electrocution. Out of 101 participants, 58 individuals met all PTSD diagnostic criteria (“PTSD” group), and 43 expirienced subthreshold PTSD symptoms but did not quantify for PTSD diagnosis (“No PTSD” group).

### Clinical instruments

PTSD symptom severity was quantified using the Clinician-Administered PTSD Scale for DSM-IV (CAPS-4)^37^, administered by trained and certified clinical interviewers. Additionally, four self-report questionnaires were administered: PTSD Checklist for DSM-IV (PCL-4)^38^ evaluating post-traumatic symptoms; Beck’s Depression Inventory (BDI)^39^ assessing current depressive symptoms; Beck’s Anxiety Inventory (BAI)^40^ measuring current anxiety symptoms; and Participants’ Clinical Global Impression Scale (CGI-P)^41^ evaluating patients’ subjective impression, on a scale between 1 (“normal feeling”) to 7 (“the worst feeling there is”). For detailed description see^36^.

### Cognitive functioning

WebNeuro^42^, an internet-based comprehensive cognitive assessment battery previously validated against traditional cognitive tests, was used to assess cognitive functioning. To standardize testing conditions, all tests were conducted in our laboratory and in Hebrew. Performance on the different tasks was calculated by the WebNeuro software that derived standardized *Z*-scores for each participant on each of the following eleven cognitive domains: motor coordination, processing speed, sustained attention, controlled attention, cognitive flexibility, response inhibition, working memory, recall memory, executive function, emotion identification, and emotional bias (see also Ben-Zion et al.^13,36^).

### Imaging data acquisition

Structural and functional scans were performed in a 3.0 Tesla Siemens MRI system (MAGNETOM Prisma, Germany), using a twenty-channel head coil, located in our lab at Tel-Aviv Sourasky Medical Center. To allow high-resolution whole-brain structural images, a T1-weighted magnetization prepared rapid gradient echo (MPRAGE) (TR/TE = 2400/2.29 ms, flip angle = 8°, voxel size 0.7 × 0.7 × 0.7 mm, FOV = 224 × 224 mm) was acquired. Functional whole-brain scan was performed in an interleaved order, using a T2*-weighted gradient echoplanar imaging pulse sequence (TR/TE = 2000/28 ms, flip angle = 90°, voxel size 2.2 × 2.2 × 2.2 mm, FOV = 220 × 220 mm, and slice thickness = 3 mm, 36 slices per volume).

### Structural imaging data analysis

Cortical reconstruction and volumetric segmentation were performed with the FreeSurfer image analysis suite^43^ (version 1.379.2.73), which is documented and freely available for download online (http://surfer.nmr.mgh.harvard.edu/). Briefly, this processing included motion correction and the averaging^44^ of multiple volumetric T1-weighted images, removal of non-brain tissue using a hybrid watershed/surface deformation procedure^45^, automated Talairach transformation, segmentation of the subcortical white matter and deep gray matter volumetric structures (including hippocampus, amygdala, caudate, putamen, and ventricles)^46,47^, intensity normalization, tessellation of the gray matter-white matter boundary, automated topology correction, and surface deformation following intensity gradients to optimally place the gray/white and gray/cerebrospinal fluid borders at the location where the greatest shift in intensity defines the transition to the other tissue class. The automatic subcortical segmentation of brain volume is based upon the existence of an atlas (“aseg”) containing probabilistic information on the location of structures^48^. The maps are created using spatial intensity gradients across tissue classes and are therefore not simply reliant on absolute signal intensity. The maps produced are not restricted to the voxel resolution of the original data. Thus they are capable of detecting submillimeter differences between groups.

### Functional imaging data analysis

Preprocessing and statistical analysis of the functional images were performed in a voxel-based approach using Statistical Parametric Mapping (SPM)^49^ version 12. In short, this process included: slice time correction, using one slice before the last as the reference slice. Head motion correction by six-parameter rigid body spatial transformations, using three translations and three rotation parameters, with the first image serving as a volume reference. A 4th degree interpolation was applied to detect and correct head motions. Functional maps were automatically co-registered to corresponding structural maps using an objective function of normalized mutual information (NMI). The complete dataset was transformed into MNI space and spatially smoothed with an isotropic 6-mm full-width at half-maximum (FWHM) Gaussian kernel.

During this scan, participants performed the Emotional Faces Matching Task^50^, which was used to evaluate their emotional reactivity. In this task, subjects were instructed to select the face/shape (located at the bottom right or bottom left of the screen) that matches the target face/shape (located at the top of the screen), as accurately and as quickly as possible. The tasks included four blocks of shapes (that were used as a baseline) and four blocks of emotional faces (angry, fearful, surprised, and neutral faces). The order of the blocks of emotional faces was counterbalanced between subjects using four different versions for this task. Both whole-brain activations and functional connectivity of the amygdala (seed region) were calculated for the following contrasts: angry faces (vs. shapes), fearful faces (vs. shapes), surprised faces (vs. shapes), and neutral faces (vs. shapes). For a full list of the functional brain measures derived from this analysis, please refer to “brain function variables” in Supplementary Table 1.

### Procedure

A member of the research team identified potentially trauma-exposed patients using the ED medical records. Within 10–14 days after ED admission, and after being discharged from the hospital, these individuals were contacted for an initial telephone screening, which was conducted by MA-level clinicians that were trained in the specific assessment tools. After obtaining verbal consent, the PCL-5 was administered to assess the risk of PTSD development. Those who met PTSD symptom criteria (except the “1-month duration” criteria) and did not meet any of the exclusion criteria (see^36^), received verbal information about the study and were invited to a clinical assessment. The latter comprised the CAPS, self-administered questionnaires (BDI, BAI, PCL, and CGI) and the WebNeuro cognitive battery. Participants were then invited to a brain imaging session that included structural and functional MRI. Each meeting (clinical assessment and MRI scan) lasted ~3 h, and both were conducted within 30 days of ED admission. The participants received financial remuneration, in accordance with the ethics committee regulations and approval.

### Statistical approach

The 3C method^35^ assumes that existing medical knowledge of a given disorder is critical but not sufficient for an accurate diagnosis. It offers to build upon and expand the current diagnostics with unsupervised data-driven methods. The 3C utilizes previously validated clinical measures, relevant to the disease diagnosis, to divide patients into homogeneous clusters based on common characteristics. It then further characterizes those groups (i.e., clusters) by exploring multi-domain potential biomarkers, which relate to the specific disorder-subtype. Previous studies using the 3C^51,52^ discovered new sub-phenotypic groups and their specific biomarkers in a large Alzheimer’s disease dataset (“ADNI”^53^).

The 3C procedure comprises three stages; categorize, cluster, and classify. During categorization, the multi-domain variables are sorted into three categories; (1) Assigned diagnosis as applied in the field. (2) Clinical measurements; variables that describe the patient’s condition and the expression of the disease (symptoms and signs). (3) Potential biomarkers; variables that could improve existing diagnostic procedures but are not currently in clinical use. Clustering includes two steps: (1) Feature Selection: A supervised selection of the most relevant clinical variables to the assigned diagnosis, based on a permissive threshold of Benjamini–Hochberg^54^ False Discovery Rate (FDR)-adjusted of *p* = 0.2 (from now on, the use of “FDR” in this manuscript will specifically refer to the BH procedure). This was done to eliminate clinical variables that are not relevant to the disease as defined by the assigned diagnosis category. Although there is no single specific FDR value recommended for this purpose, and different values chosen may affect the clusters formed, the value of *p*_*FDR*_ = 0.2 is often used in genomics and in other a screening efforts preceding analyses. (2) Unsupervised clustering: Utilizing the selected clinical measurements was performed using k-medoids with Manhattan distance metrics. This allowed us to discover data-driven homogeneous clusters that are related to existing diagnostics (i.e., dividing participants into subtypes based on commonly used variables). Nevertheless, these clusters are not limited to formal symptom-based PTSD diagnosis (as indicated by CAPS), but rather capture the actual richness of phenotypical features associated with post-traumatic psychopathology within a dataset. Before clustering, the optimal number of clusters was determined based on two metrics: gap statistics^55^ and silhouette^56^. Lastly, classification includes characterization of the clusters based on the objective potential biomarkers, via three distinct approaches: importance analysis (mean decrease GINI^57^); classification tree; and a marginal one-way analysis of variance (ANOVA) between clusters for each potential biomarker.

### Algorithms, codes, and software

Algorithms and codes were performed using R software version 3.4.4. Data imputation was performed using the 5-nn method in order to deal with missing data (<1% of the full dataset). Variables were monotonically transformed to gain symmetry when needed, using a semi-automated Shiny App^58^. Importance, measured as the marginal loss of classification accuracy for each variable by randomly permuting it on the out-of-bag validation set, was calculated using the {randomForest} R package^57^. R package {cluster}^59^ was used for clustering, and R package {rpart} was used for classification and regression trees (CART).

## Results

During categorization, features were divided into the following three distinct categories: assigned diagnosis was based on the CAPS-4 total scores; clinical measurements included the total scores of the four self-report questionnaires (PCL, BDI, BAI, and CGI); potential biomarkers included 11 standardized total scores obtained from computerized cognitive testing, 192 features from structural imaging (volumes and thickness of subcortical and cortical areas), and 48 features extracted from fMRI during the emotional faces matching task (whole-brain activations and functional connectivity of left and right amygdala during the task). For a full list of the features used for this analysis, see Supplementary Table 1.

For clustering, the clinical measurements that were found highly correlated with PTSD symptom severity (as indicated by CAPS-4 total scores) were used: PCL, BDI, BAI, and CGI (based on the ad-hoc threshold, *p*_*FDR*_ < 0.2). Participants were divided into an optimal number of two clusters, based on both gap statistics^55^ and silhouette^56^ methods, which presented the best separation on all four clinical measurements (PCL, BDI, BAI, and CGI).

**Fig. 1:**
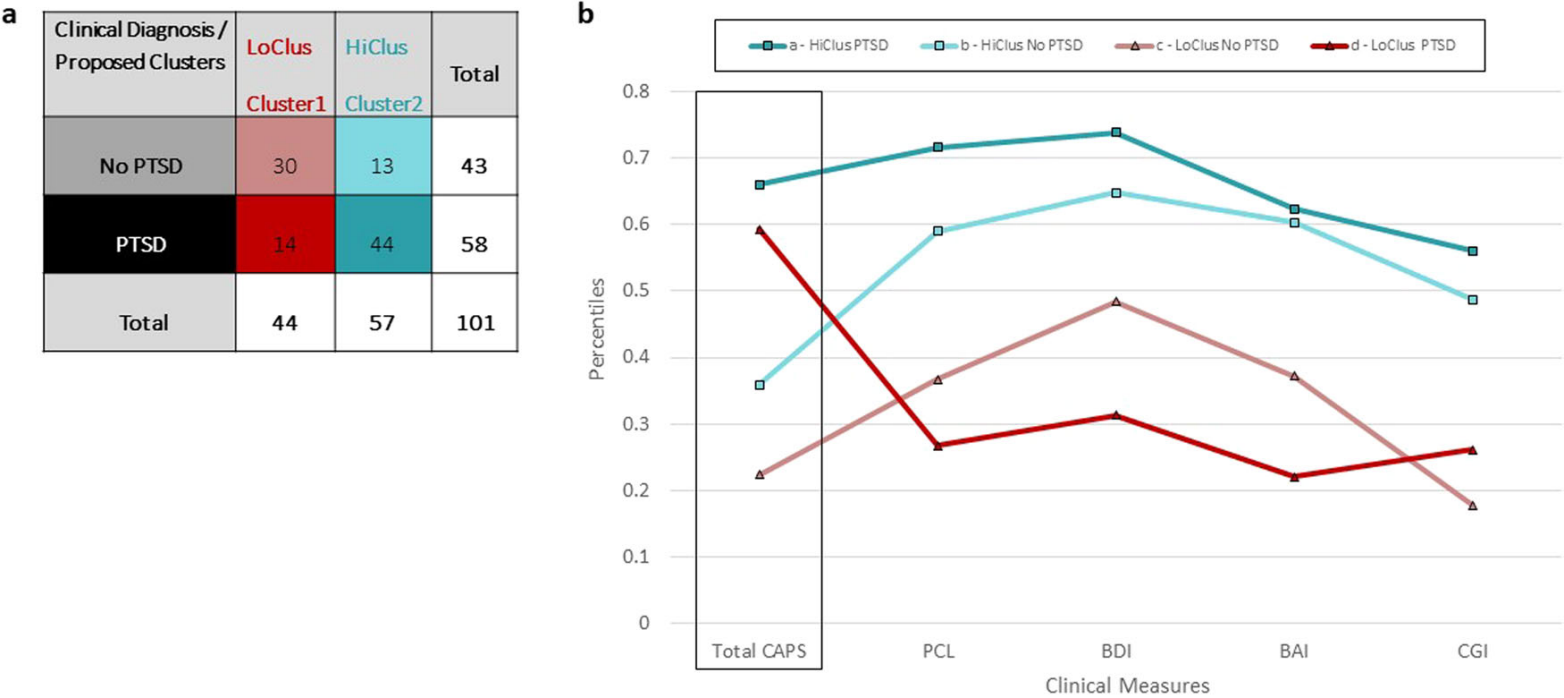
Parallel coordinates plot of assigned diagnosis and clinical measurements. **a** Confusion matrix of PTSD diagnosis versus proposed clusters. Table rows represent individuals’ current clinical DSM-based PTSD diagnosis (PTSD/No PTSD), while the columns represent the two proposed clusters (Cluster1 = LoClus = low-symptomatic cluster/Cluster2 = HiClus = high-symptomatic cluster). This division to clinical diagnosis and proposed clusters created four different groups, colored according to the four lines they represent in part b of this figure. **b** Parallel coordinates plot of the different groups. The *X*-axis depicts the four clinical measurements on which the clusters were built (BDI, BAI, PCL, and CGI), as well as the assigned diagnosis (total CAPS score), while the *Y*-axis depicts their percentiles (standardized values, ranging from 0 to 1). The figure presents the means of each variable for each of the four groups, created by the division to two clusters (HiClus – turquoise, squares; LoClus – red, triangles) and two clinical DSM-based diagnosis (PTSD – darker colors; No PTSD – lighter colors). CGI = Total Score of Clinical Global Impression Scale Questionnaire, PCL = Total Score of PTSD Checklist Questionnaire, BAI = Total Score of Beck Anxiety Inventory Questionnaire, BDI = Total Score of Beck Depression Inventory Questionnaire.

To test the association between these two clusters (representing high and low “disease load”) and the formal clinical PTSD diagnosis (PTSD or no-PTSD, according to CAPS-4), a two-sample test for equality of PTSD proportions between the two clusters was conducted. Results revealed a significant link between the proposed clusters and PTSD dichotomous diagnosis *(Z* = 4.57*, p* < 0.001; see table in Fig. 1a). Accordingly, cluster 1 will now be referred to as the “low-symptomatic” cluster (LoClus, also corresponding to low severity PTSD), and cluster 2 as the “high-symptomatic” cluster (HiClus, also corresponding to high severity PTSD severity). Furthermore, a one-way ANOVA showed a significant difference between the two clusters in continuous PTSD symptom severity (i.e., CAPS-4 total scores) (*F*_1,99_ = 35.47, *p* < 0.001). Results indicated that individuals belonging to the HiClus had significantly higher CAPS-4 total scores and proportion *(P* = 0.77, *M* = 61.91 ± 18.16) compared to those belonging to the LoClus (*P* = 0.32, *M* = *37.45* ± 23.12; *p* < 0.001; see Supplementary Fig. 1). It is important to note that these clusters do not reflect a new diagnosis of PTSD, but rather a means to an end to find potential biomarkers. As mentioned, these clusters were found to correlate with PTSD clinical diagnosis and severity, but were not identical to it, and therefore represent clusters of “disease load” or “severity subtypes”.

The differences in the assigned diagnosis (total CAPS score) and clinical measurements (BDI, BAI, PCL, and CGI) between the proposed clusters (HiClus, LoClus) and the DSM-based diagnosis (PTSD, No PTSD) are presented in Fig. 1b. Examining the differences between HiClus (light and dark turquoise, groups a & b) and LoClus (light and dark red, groups c & d), participants of the HiClus showed an average higher severity on all four clinical measurements (representing symptoms of PTSD, anxiety and depression, as well as general subjective feeling), compared to those of the LoClus. Examining the two groups within the HiClus, as expected, individuals diagnosed with PTSD (HiClus PTSD, group a, dark turquoise) showed higher severity scores on all four clinical measurements, compared to those not diagnosed with PTSD in this cluster (HiClus No PTSD, group b, light turquoise). Examining the two groups within the LoClus however, surprisingly, individuals diagnosed with PTSD (LoClus PTSD, group d, dark red) showed lower severity on three (out of four) clinical measurements (BDI, BAI, and PCL), compared to those not diagnosed with PTSD in this cluster (LoClus No PTSD, group c, light red). Nevertheless, this LoClus PTSD group (group d, dark red) exhibited higher total CAPS scores, compared to both LoClus No PTSD and HiClus No PTSD groups (group b and c, respectivley). This demonstrates the added value of our division to clusters, exmaining a braoder array of clinical measures, rather than relying only on CAPS total scores.

**Fig. 2:**
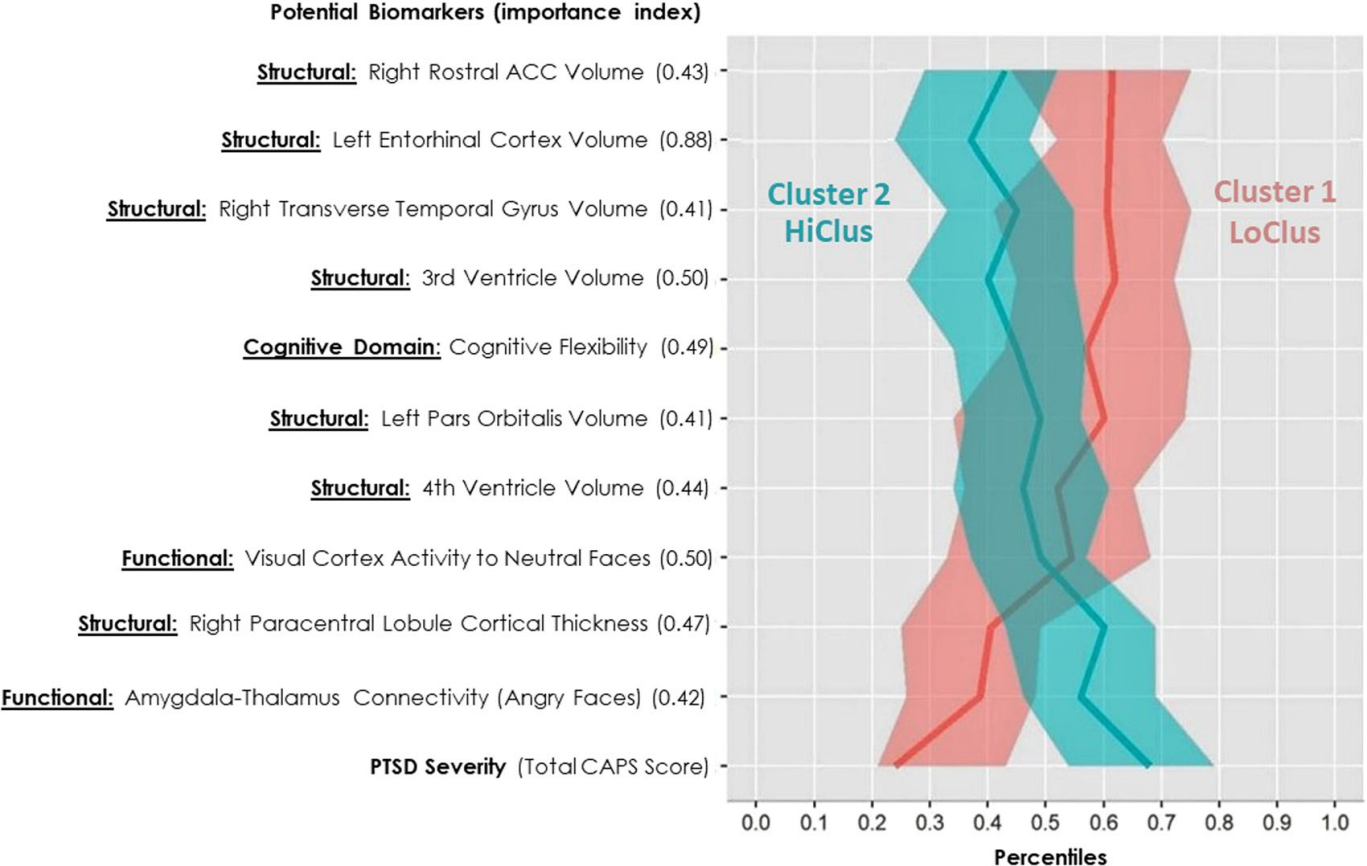
Parallel coordinates plot of potential biomarkers. The *Y*-axis depicts the top 10 most important potential biomarkers in classifying the two clusters, together with their mean decrease GINI measure (i.e. importance index). The domain of each biomarker is presented as a prefix – structural brain measurements (“structural”), functional brain measurements (“functional”), and cognitive domains. Average CAPS-4 total scores is presented for both “low-symptomatic” cluster (cluster 1, LoClus, red) and “high-symptomatic” cluster (cluster 2, HiClus, turquoise). The medians of 400 Bootstrap samplings were drawn, and their median and 0.025 and 0.975 percentiles are plotted per cluster.

During the classification stage, objective variables that differentiate between the classes, hence could serve as potential biomarkers, were examine by using a mean decrease importance index (GINI). The most significant potential biomarkers associated with the resulted clusters included left entorhinal cortex (EC) volume (importance = 0.884), cognitive flexibility (importance = 0.487), rostral anterior cingulate cortex (rACC) volume (importance = 0.429), and average amygdala functional connectivity with the thalamus while watching angry faces vs. shapes (importance = 0.419). The top ten potential biomarkers for the clustering are presented in Fig. 2.

**Fig. 3:**
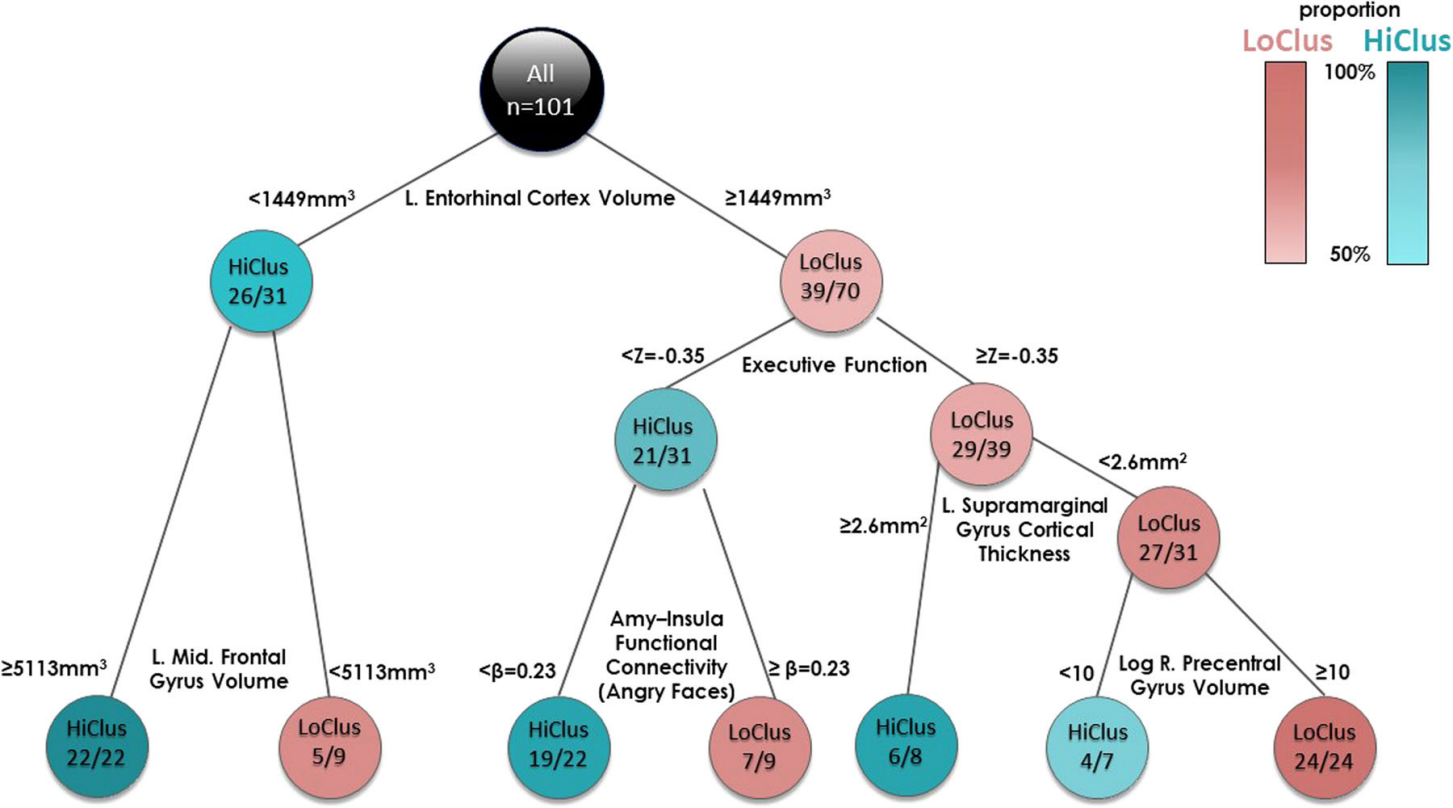
Classification tree based on the two clusters. The classification tree depicts variables important for the division of the participants to the two clusters, starting from the most important one at the top of the tree (left entorhinal cortex volume). Each block is labeled either HiClus or LoClus, indicating whether most of the subjects in that block belong to the HiClus or the LoClus (turquoise or red) and their proportion (from 50%=lighter colors to 100%=darker colors, see color bar at the top right). Furthermore, each block shows the number of subjects belonging to the dominant cluster (either HiClus or LoClus), and the total number of subjects in that specific block. Inspecting the top block for example, 70 out of 101 participants had left EC volume ≥1449 mm^3^, out of which 39 belonged to the LoClus. The other 31 participants had a left EC volume <1449 mm^3^, out of which the most (*n* = 26) belonged to the HiClus.

To further characterize patients within each cluster according to the identified biomarkers, classification tree was built (see Fig. 3). Results indicated that left hemisphere EC volume had the greatest influence on clustering – 70 out of 101 participants had left EC volume equal to or >1449 mm^3^, of which there was almost an equal distribution between the two clusters (56%/44%). The other 31 participants had a left EC volume <1449 mm^3^, of which the vast majority (84%) belonged to HiClus; indicating that subjects with lower EC volume were more likely to belong to the HiClus. Further down on the left branch of the tree, HiClus subjects had larger left caudal middle frontal gyri volume. Down the right branch of the tree, high executive functioning was more associated with LoClus, and vice versa. Further down, low supramarginal gyrus cortical thickness, together with high paracentral volume, were related to LoClus. In contrast, low executive functions with low functional connectivity between the amygdala and the left insula while watching fearful faces vs. shapes was strongly related to HiClus.

Finally, a marginal one-way ANOVA was conducted on the potential biomarkers with HiClus/LoClus as the dependent variable. Benjamini–Hochberg adjustment to control the FDR at level 0.05, yielded no significant difference for any potential biomarker between HiClus/LoClus, FDR-corrected (partly due to hundreds of *p*-values that were adjusted).

To assess robustness and consistency within this sample, the procedure detailed above was cross-validated. The 3C method was performed on a different percentage, *P* = 20%, 30%, …, 90% of all participants to be used as a training sample. Two clusters were chosen and classification tree for these two clusters were built, all on the training sample. The other 100%-P individuals were used as a validation sample, and were classified to the new clusters based on the newly created classification tree. We report the mean percentage (and SD) of individuals classified by the smaller trees into the original full data built clusters, after matching the new clusters to the overall ones based again on the validation test. The results across 1000 iterations for each percentage *P* is presented in Table 1. For example, when the 3C algorithm was based only on a training set of 20% of the subjects, 83% of the validation sample were classified to the original high and low severity clusters (on average). Incidentally, we always used two clusters, but when the algorithm was allowed to estimate optimally the number of clusters, two clusters were chosen in 82% of the iterations, the rest requiring 3 or more clusters.

**Table 1:** Cross-validation results.

*P*	20%	30%	40%	50%	60%	70%	80%	90%
Mean (%)	83	86	86	88	89	90	90	95
SD (%)	10	7	10	9	7	7	6	7

The table presents the mean percentage and standard deviation (SD) of subjects who were correctly classified (according to the results of the 3C methodology based on all subjects).

For each P, *n* = 1000 iterations were performed.

## Discussion

This work illustrates the principles and results of a novel three-staged hybrid analytic approach (3C). First, we demonstrated a classification of recent trauma survivors into two different subtypes, related to PTSD symptom severity (HiClus & LoClus). Importantly, these subtypes differ from formal PTSD diagnosis (i.e., CAPS scores), as they encompass additional symptoms’ phenotypes of depression and anxiety. Second, a wide range of potential biomarkers, derived from cognitive and neural domains, were screened computationally, and yielded an effective separation between these new subtypes. Third, we demonstrated the usage of such potential biomarkers to form an individual-based diagnosis of PTSD based on multi-domain objective measurements.

### Hybrid approach for PTSD classification

The 3C “hybrid” data- and theory-driven approach combined current diagnostic-based categorization, symptom severity-based unsupervised clustering, and data-driven classification using a wide range of psychological, cognitive, and neural potential biomarkers. Unlike supervised machine learning models that “flatten” information and dimensions into one data matrix, the 3C method uniquely and in a stepwise manner combines current information from PTSD diagnostics with two layers of data-driven exploration: a broad picture of clinical symptoms presented shortly after trauma (including those derived from other clinical categories) and concurrently-recorded objective measurements of cognitive functioning, brain structure and function. This practice, therefore, does not impose an extraneous construct (DSM-based diagnosis) as an only outcome of interest, but instead utilizes the richness of phenotypical features associated with post-traumatic psychopathology. In our case, we utilized self-reports of PTSD, depression, anxiety and global impression (PCL, BDI, BAI, and CGI; respectively), to expand and enrich DSM-based diagnosis of PTSD. This was clearly demonstrated in Fig. 1b, where a subgroup of 14 individuals (group d, dark red) showed high total CAPS scores, while exhibiting the lowest severity in other clinical measurements (self-reported PTSD, depression and anxiety). This is one of the added values of our hybrid 3C approach, going beyond the DSM formal diagnostic categorization.

### Potential biomarkers for PTSD subtypes

The potential biomarkers revealed by the 3C analytic approach are in line with previously documented neural and cognitive correlates of PTSD, providing a framework for an early objective mechanism-based and clinically meaningful categorization of trauma survivors’ psychopathology. Accordingly, the potential biomarkers included features obtained from both neuroimaging and cognitive testing.

The structural brain feature with the greatest influence on PTSD classification was entorhinal cortex (EC) volume (both according to importance analysis and classification tree, see Figs. 2 and 3); lower EC volume was associated with higher post-traumatic stress symptoms severity load. The EC plays an important role in memory, a key feature of post-traumatic psychopathology, as uncontrolled recall of the traumatic event determines symptom severity^60–62^. Another structural feature of importance to classification was the volume of the rostral anterior cingulate cortex (rACC); lower rACC volume was associated with higher PTSD severity. Indeed, rACC volume has previously been associated with PTSD^23,63,64^, and was shown to predict cognitive-behavioral treatment response^65^; suggesting its potential as a guide for early mechanism-based intervention.

Of note, our classifier did not identify several structural abnormalities found in previous cross-sectional PTSD studies^6,14,23,27^. This includes the most replicated finding of small hippocampus volume^66^, but also abnormal amygdala volume, insular cortex, medial, and dorsal prefrontal cortices (mPFC and dlPFC respectively)^17,20,22,67–69^. This could stem from our classifier identifying early-stage biomarkers related to high and low disease-load, rather than DSM-based dichotomized PTSD diagnosis. Furthermore, most of the above-mentioned structural abnormalities were detected in individuals suffering from chronic PTSD, and not in individuals in an early stage after trauma. Here we identified structural abnormalities within 1 month after trauma as related to symptom severity. Since major changes in gray matter volume within this time frame are less likely to occur^70^, these abnormalities might be early predisposing risk factors for chronic PTSD development. Future studies in populations prone to trauma exposure with longitudinal measurement could shed more light on the causal inference of our findings^71–73^.

From the functional neuroimaging domain, amygdala functional connectivity with both the insula and the thalamus was found to be particularly important for classification (both according to importance analysis and classification tree, see Figs. 2 and 3). Aberrant connectivity of the amygdala with other structures is consistent with previous studies, and abnormal amygdala activation had been hypothesized to contribute to PTSD pathophysiology^74–76^. Moreover, thalamic dysfunction has been found in patients with PTSD, suggesting its role in the disorder’s psychopathology^77,78^. Therefore, although neuroimaging studies have implicated several functional brain abnormalities in the pathophysiology of PTSD, our computational analysis showed that some of these abnormalities are involved in the PTSD severity subtype in the early aftermath of trauma.

The most significant cognitive related potential biomarkers were indices of cognitive flexibility (according to importance analysis, see Fig. 2) and executive function (according to classification tree, see Fig. 3). Indeed, meta-analyses regarding the role of cognitive functions in PTSD consistently show an impaired ability in executive functioning, including cognitive flexibility (the ability to switch between two different tasks or strategies) among PTSD patients^79,80^. More so, cognitive flexibility shortly after trauma was shown to be a significant predictor of PTSD severity 1 year later, and ameliorating it by a cognitive intervention was associated with better treatment outcomes^13^. Altogether, implying the role of cognitive flexibility in early recovery following trauma exposure. One possibility is that intact cognitive flexibility enables the individual to better differentiate between threat-related and neutral situations, thus assisting in the extinction of fear-motivated learning, a core-element in PTSD recovery^81^.

### Clinical considerations

Our 3C approach revealed two PTSD subtypes (classes) in recent trauma survivors, correlated with high and low clinical severity, according to total CAPS scores, and across all CAPS subscales (re-experiencing, avoidance, negative alterations in cognitions and mood, and alternations in arousal and reactivity). Our analysis did not find classes representing different clinical subtypes, such as greater dissociation or avoidance. This could be because the 3C is based on a given set of subjective clinical measures (BDI, BAI, PCL, and CGI), and classification was further based on predetermined objective measures of potential biomarkers. Indeed, in an effort to account for the heterogeneity in PTSD expression, several studies attempted to characterize different clinical subtypes of the disorder. For example, an externalizing subtype characterized by low constraint and high negative emotionality, compared to an internalizing cluster with high negative emotionality and low positive emotionality^82–84^; or a dissociative subtype for patients with PTSD and de-personalization and/or de-realization symptoms, introduced by the fifth edition of the DSM^85–87^. Furthermore, the cognitive and neural biomarkers presented here are not yet biologically validated markers (see suggested strategy for the development of biomarker tests for PTSD^88^), and therefore could only be regarded as “potential biomarkers”.

It is overall acknowledged that a larger dataset (i.e., more measures and more participants), could allow for the identification of unique clinical classes of different symptomatic subtypes, possibly more than two. More so, adding longitudinal measures from different time-points following trauma may reveal classes corresponding to PTSD clinical trajectories. This may be crucial for the identification of individuals at risk for developing PTSD, as well as providing appropriate early-stage treatment.

## Conclusion

Our study implemented an innovative semi-unsupervised computational approach that unveiled novel variables correlated with the morbidity classification of recent trauma survivors. The method utilized current DSM-based PTSD diagnostic categories and other clinical severity measures of depression and anxiety, as well as a classification of cognitive and neural potential biomarkers. Intriguingly the two subtypes of PTSD severity were also associated with known neurocognitive mechanisms underlying post-traumatic stress symptoms. Our results point to an alternative approach for identifying objective variables linked to PTSD severity subtypes (high and low), based on testing within a single session shortly after exposure to trauma. If successful, this objective computational classification may further guide mechanism-driven diagnosis and interventions for PTSD (e.g., cognitive remediation or neuromodulation treatments). If performed on a broader data set and with more clinical measures and potential biomarkers, this hybrid approach may refine post-traumatic diagnostic subtypes, playing an important role in the clinical management of recent trauma survivors.

## Supplementary information

Supplementary Materials
