# Supplementary material for: Multi-domain potential biomarkers for post-traumatic stress disorder (PTSD) severity in recent trauma survivors
Source: Transl Psychiatry. 2020 Jun 27;10:208. doi: 10.1038/s41398-020-00898-z (PMC7320966; doi:10.1038/s41398-020-00898-z)

Supplementary Materials

Supplementary Table 1. Neurocognitive Features

Supplementary Figure 1. Difference in PTSD Symptom Severity between Clusters

Supplementary Table 1

Neurocognitive Features. This table describes the 256 neurocognitive features that were used for the 3C analysis. These variables are sorted by domain: clinical (n=5), cognitive (n=11), brain structure (n=192), and brain function (n=48).

**Clinical Variables (n=5):**<br>Total scores of CAPS-4 and the following self-report questionnaires: PCL, BAI, BDI, CGI.
**Cognitive Variables (n=11):**<br>Standardized Z-scores of the following domains: motor coordination, processing speed, sustained attention, controlled attention, cognitive flexibility, response inhibition, working memory, recall memory, executive function, emotion identification, and emotional bias.
**Brain Structure Variables (n=192):**<br>**A.** Volumes (mm^3) of the following subcortical areas (separately for left and right hemisphere): lateral ventricle, inferior lateral ventricle, cerebellum white matter, cerebellum cortex, thalamus, caudate, putamen, pallidum, 3rd ventricle, 4th ventricle, 5th ventricle, brain stem, hippocampus, amygdala, CSF, accumbens, ventral diencephalon cortex, vessel, choroid plexus, optic chiasm, corpus callosum (posterior, mid-posterior, central, mid-anterior, anterior), subcortical gray volume, total gray volume, total intracranial volume.<br>**B.** Volumes (mm^3) of the following cortical areas (separately for left and right hemisphere): banks of superior temporal sulcus, caudal anterior cingulate cortex, caudal middle frontal cortex, cuneus, entorhinal cortex, frontal pole, fusiform gyrus, inferior parietal cortex, inferior temporal cortex, insula, isthmus cingulate cortex, lateral occipital cortex, lateral orbitofrontal cortex, lingual cortex, medial orbitofrontal cortex, middle temporal cortex, paracentral lobule, parahippocampal gyrus, inferior frontal gyrus (pars opercularis, pars orbitalis, pars triangularis), peri-calcarine cortex, post-central cortex, pre-central cortex, posterior cingulate cortex, precuneus, rostral anterior cingulate cortex, rostral middle frontal cortex, superior frontal cortex, superior parietal cortex, superior temporal cortex, supramarginal gyrus, temporal pole, transverse temporal gyrus.<br>**C.** Cortical thickness (mm^2) of the following cortical areas (separately for left and right hemisphere): banks of superior temporal sulcus, caudal anterior cingulate cortex, caudal middle frontal cortex, cuneus, entorhinal cortex, frontal pole, fusiform gyrus, inferior parietal cortex, inferior temporal cortex, insula, isthmus cingulate cortex, lateral occipital cortex, lateral orbitofrontal cortex, lingual cortex, medial orbitofrontal cortex, middle temporal cortex, paracentral lobule, parahippocampal gyrus, inferior frontal gyrus (pars opercularis, pars orbitalis, pars triangularis), peri-calcarine cortex, post-central cortex, pre-central cortex, posterior cingulate cortex, precuneus, rostral anterior cingulate cortex, rostral middle frontal cortex, superior frontal cortex, superior parietal cortex, superior temporal cortex, supramarginal gyrus, temporal pole, transverse temporal gyrus.
**Brain Function Variables (n=48):**<br>**A.** Average brain activations (FWE<0.05, whole-brain corrected) in the amygdala (left & right), hippocampus (left & right), fusiform gyrus (left & right), and visual area, in response to the following contrasts: angry faces (vs. shapes), fearful faces (vs. shapes), surprised faces (vs. shapes), and neutral faces (vs. shapes).<br>**B.** Average functional connectivity (PPI) between the amygdala (left & right) and thalamus, insula, ventral anterior cingulate cortex, in response to the following contrasts: angry faces (vs. shapes), fearful faces (vs. shapes), surprised faces (vs. shapes), and neutral faces (vs. shapes).

Supplementary Figure 1

Difference in PTSD Symptom Severity between Clusters. Boxplots depicting CAPS-4 total scores (Y-axis) for each of the two clusters (cluster 1 and 2, LoClus & HiClus, respectively) extracted in the clustering stage of the analysis.

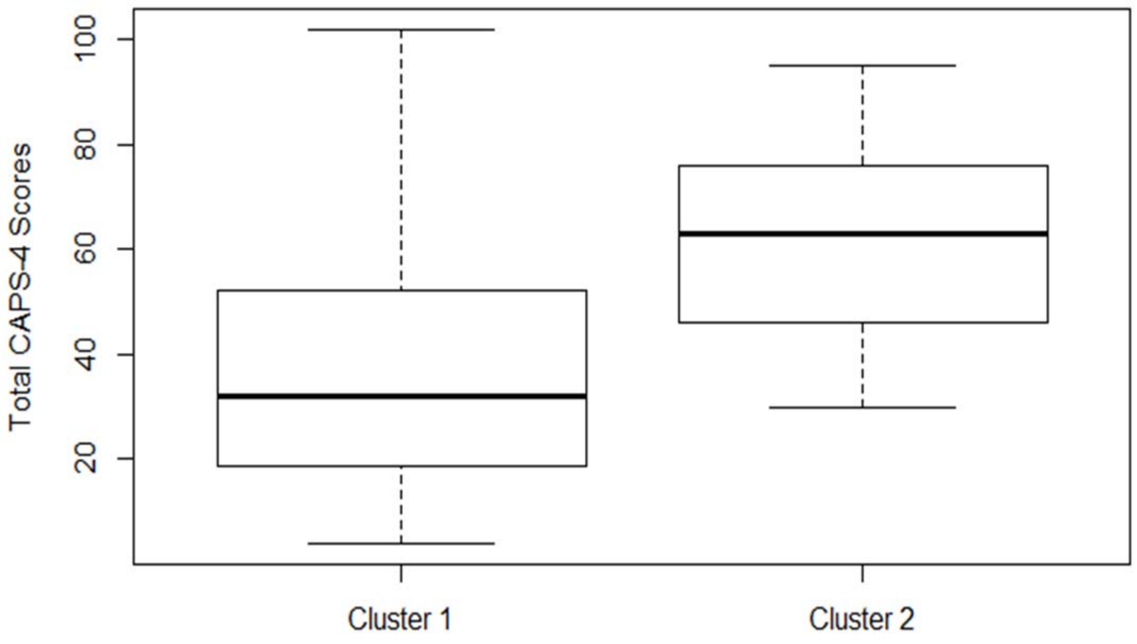

Supplement: Supplementary file 1 — Supplementary Materials [file 41398_2020_898_MOESM1_ESM.pdf]
